# Deficiencies in one-carbon metabolism led to increased neurological disease risk and worse outcome: homocysteine is a marker of disease state

**DOI:** 10.3389/fnut.2024.1285502

**Published:** 2024-02-21

**Authors:** Sanika M. Joshi, Nafisa M. Jadavji

**Affiliations:** ^1^College of Osteopathic Medicine, Midwestern University, Glendale, AZ, United States; ^2^Department of Biomedical Sciences, Midwestern University, Glendale, AZ, United States; ^3^Department of Child Health, College of Medicine – Phoenix, University of Arizona, Phoenix, AZ, United States; ^4^College of Veterinary Medicine, Midwestern University, Glendale, AZ, United States; ^5^Department of Neuroscience, Carleton University, Ottawa, ON, Canada

**Keywords:** homocysteine, one-carbon metabolism, cognitive decline, ischemic stroke, folate, folic acid

## Abstract

Elevated plasma homocysteine levels have been identified as a significant, independent risk factor for the development of cognitive decline including Alzheimer’s disease. While several studies have explored the link between homocysteine and disease risk, the associations have not been entirely clear. Elevated levels of homocysteine serve as a disease marker and understanding the underlying cause of these increased levels (e.g., dietary or genetic deficiency in one-carbon metabolism, 1C) will provide valuable insights into neurological disease risk and outcomes. Previous cell culture experiments investigating the mechanisms involved used ultra-high levels of homocysteine that are not observed in human patients. These studies have demonstrated the negative impacts of ultra-high levels of homocysteine can have on for example proliferation of neuroprogenitor cells in the adult hippocampus, as well as triggering neuronal apoptosis through a series of events, including DNA damage, PARP activation, NAD depletion, mitochondrial dysfunction, and oxidative stress. The aim of this mini-review article will summarize the literature on deficiencies in 1C and how they contribute to disease risk and outcomes and that homocysteine is a marker of disease.

## 1 Introduction

The history of homocysteine dates back to approximately 90 years ago when it was first identified by scientists Butz and du Vigneaud during their investigation of the decomposition products of the amino acid methionine (1). Initially, homocysteine was considered a relatively unremarkable sulfur-containing amino acid. However, as research progressed, it became evident that homocysteine plays a crucial role in linking sulfur, methionine, and one-carbon (1C) metabolism (1).

1C metabolism is a complex network of biochemical reactions involving various B-vitamins, which act as cofactors, precursors, and substrates for numerous biological processes (2). In 1C, methionine, an essential amino acid obtained from dietary sources, is processed through the methylation cycle, leading to the formation of homocysteine (2). In turn, homocysteine can either be remethylated back to methionine or catabolized through the transsulfuration pathway to form cysteine and other metabolites. The methylation cycle is particularly important as it involves the activation of methionine into *S*-adenosylmethionine (SAM), a universal methyl donor utilized by DNA, RNA, histone, and protein methyltransferases. Methylation is critical for various cellular processes, including protein–protein interactions and epigenetic regulation, which play important roles in embryonic development, cognitive function, and hematopoiesis.

Perturbations in the uptake and homeostasis of B-vitamins, leading to deficiency or excess of 1C metabolism intermediates, can have significant health implications. Defects in enzymes involved in homocysteine metabolism can led to severe genetic disorders known as “homocystinurias” (1). The discovery of homocystinuria, an inborn error of metabolism disorder, and the identification of severe genetic defects in enzymes involved in homocysteine metabolism, provided important insights into the role of homocysteine in human health (1). This led to the development of laboratory assays for detecting mild to moderate elevation of blood homocysteine concentrations, allowing for further research into its associations with various diseases (1).

Seshadri et al. (3) was the pioneering study that initially described the significance of homocysteine as a modifiable risk factor. This study aimed to explore the relationship between elevated plasma homocysteine levels and the risk of dementia and Alzheimer’s disease (3). The research involved 1,092 subjects without dementia, drawn from the Framingham Study, who had both baseline and previous homocysteine measurements (3). Over an eight-year follow-up period, dementia emerged in 111 individuals, including 83 diagnosed with Alzheimer’s disease (3). The study’s findings reveal that an increased plasma homocysteine level stands as a robust independent risk factor for the development of dementia and Alzheimer’s disease (3). This association displayed a consistent positive correlation, whereby higher homocysteine levels were linked to an escalated risk (3). Importantly, the correlation between homocysteine levels and dementia remained robust even after accounting for various confounding factors such as age, gender, genetics, vitamin levels, and other established dementia risk markers (3). This independence emphasized the significance of homocysteine as a potential modifiable risk factor that could be targeted for preventive interventions (3).

The implications of Seshadri et al. (3) pioneering study have reverberated across medical research. It spurred the call for comprehensive investigations involving diverse cohorts and rigorous clinical trials, aimed at establishing the causality between homocysteine levels and the risk of dementia. By unraveling the underlying mechanisms, these inquiries could potentially pave the way for targeted strategies to mitigate the risk of these debilitating conditions. It is noteworthy that a multitude of studies have illuminated the associations between elevated homocysteine levels and susceptibility to a wide array of health conditions such as heart disease, stroke, dementia, renal disease, thyroid disease, and complications during pregnancy. Additionally, the advent of technological advancements has facilitated the execution of large-scale epidemiological studies, between elevated homocysteine levels and a spectrum of neurodegenerative and cerebrovascular diseases.

The aim of this mini review is to summarize the literature on deficiencies in 1C metabolism and how they contribute to neurological disease risk and outcomes. Additionally, the review will focus on the role of homocysteine as a marker of disease, aiming to challenge the notion that elevated homocysteine levels contribute to disease. We have critically analyzed existing literature to present a comprehensive argument that hyperhomocysteinemia provides a limited perspective and is not the sole determinant of disease. We believe that understanding the complex interactions between B-vitamins and homocysteine metabolism is essential for maintaining cellular health and preventing potential health issues associated with abnormal homocysteine levels. Ultimately, the associations between homocysteine levels and various health outcomes underscore the importance of continuous research in this area, striving to enhance our understanding of human health and disease.

## 2 Cognitive decline

The hypothesis that lowering homocysteine levels through B-vitamin treatment can slow cognitive decline and prevent dementia has been extensively studied in various clinical trials and meta-analyses. The folic acid and carotid intima-media Thickness (FACIT) and the VITACOG studies, showed that B-vitamin supplementation could slow cognitive decline and reduce brain atrophy in specific populations with elevated homocysteine levels, such as individuals with mild cognitive impairment (MCI) (4). The VITACOG Trial also suggested interactions with other risk factors like omega-3 fatty acids and aspirin use (4). Overall, the results emphasize the importance of considering individual risk factors and appropriate timing for interventions in B-vitamin supplementation trials for cognitive decline and dementia prevention.

A meta-analysis of eight cohort studies found a significant association between higher serum homocysteine levels and an increased risk of dementia. For every 5 mmol/L increase in homocysteine, the risk of dementia increased by 50% (5). Conversely, a 3 mmol/L decrease in homocysteine (possible through folic acid and vitamin B12 treatment) was associated with a 22% reduction in dementia risk (5). The study suggests a potential link between elevated homocysteine and dementia, but causality could not be established. Another study which explored the association between elevated homocysteine levels and markers of neurodegeneration in an elderly non-demented Asian population revealed that higher homocysteine levels were linked to white matter atrophy, parietal cortical thinning, and smaller volumes in subcortical structures, such as the thalamus, brainstem, and accumbens (6). These results are consistent with previous studies showing similar associations in individuals with Alzheimer’s disease and mild cognitive impairment (6). However, this study did not find a significant association between homocysteine and hippocampal volume, which differed from previous reports in Caucasian cohorts (6). The study suggests that homocysteine may have a role in the early stages of cognitive impairment and neurodegeneration.

Elevated homocysteine levels are associated with an increased risk of cognitive decline and dementia, and B-vitamin treatment to lower homocysteine shows promise in mitigating these risks. While the exact mechanisms and causal relationships require further investigation, addressing elevated homocysteine levels through B-vitamin supplementation could have significant implications for dementia prevention and management.

## 3 Ischemic stroke

The role of elevated serum homocysteine concentrations (hyperhomocysteinaemia) in the risk of ischemic stroke has spurred a notable controversy within medical research. Central to this debate is whether interventions aimed at normalizing hyperhomocysteinaemia, such as B vitamin supplementation, can effectively reduce the risk of stroke. The China Stroke Primary Prevention Trial, offered insights into the potential effects of folate on stroke prevention that extend beyond homocysteine reduction (7–9). However, this study’s methodology was critiqued for overlooking factors such as smoking, other medications, and the reliability of stroke diagnoses. The Vitamin Intervention for Stroke Prevention (VISP) study examined the potential benefits of high-dose folic acid, vitamin B6, and vitamin B12 on recurrent stroke risk (10). However, despite the reduction in total homocysteine levels, this study did not find a significant difference in outcomes between high-dose and low-dose supplementation groups. A perspective article by Spence in 2007 delved into the connection between elevated homocysteine levels and the risk of different types of strokes (11). It reanalyzed the VISP trial, highlighting issues with trial design and suggesting that stroke and myocardial infarction have distinct pathogenesis origins (11). Subgroup analyses supported the potential efficacy of vitamin therapy in reducing stroke risk (11).

Additionally, investigations into genetic variants and their impact on homocysteine levels and recurrent stroke risk were conducted. The study analyzing transcobalamin variant effects revealed associations between genetic variations and homocysteine levels, as well as recurrent stroke risk (12). Furthermore, the interpretation of a genetic variant in the MTHFR gene as a significant stroke risk factor, emphasizing the intricate interplay of genetic variants and other influencing factors in determining stroke risk was thought to be too simplified an explanation by Finsterer (13).

Moreover, the oversimplification of hyperhomocysteinaemia as having a universally pathogenic effect is questioned. Instead, more comprehensive research is necessary to establish a clearer understanding of the relationship before advocating broad interventions to normalize homocysteine levels for reducing stroke risk. In conclusion, the discussed studies collectively highlight the intricate nature of the relationship between elevated homocysteine levels and stroke risk. The ongoing debate and varying study outcomes underscore the need for more comprehensive research to elucidate the true impact of hyperhomocysteinaemia on stroke risk and to guide effective preventive strategies.

Another study aimed to investigate the association between blood homocysteine concentration, the methylenetetrahydrofolate reductase (MTHFR) C677T polymorphism, and folic acid intervention in relation to stroke risk among hypertensive individuals. Data from 20,424 participants in the China Stroke Primary Prevention Trial were analyzed (7). In the control group, elevated baseline homocysteine levels were linked to an increased risk of first stroke among participants with the CC/CT genotype of the MTHFR polymorphism (7). However, this association was not observed among participants with the TT genotype, indicating a significant interaction between the gene and homocysteine. In contrast, in the folic acid intervention group, homocysteine did not significantly impact stroke risk regardless of genotype. Folic acid intervention was found to significantly reduce stroke risk in participants with CC/CT genotypes and high homocysteine levels (7).

This finding challenges the notion that elevated homocysteine levels are the sole cause of stroke. The interaction between the MTHFR genotype and homocysteine levels suggests that genetic factors play a crucial role in the relationship between homocysteine and stroke risk (7). Additionally, the effectiveness of folic acid intervention in reducing stroke risk among individuals with specific genotypes highlights the potential impact of nutritional interventions in modulating stroke risk, independent of homocysteine levels.

Furthermore, the study by Zhao et al. (14) provides insights into the impact of folic acid deficiency on neuronal autophagy and brain injury in rats subjected to focal cerebral ischemia–reperfusion. This study emphasizes the role of folic acid in neuronal health and its potential implications for stroke prevention. The results suggest that folic acid deficiency exacerbates autophagic activation and worsens brain damage following cerebral ischemia–reperfusion, indicating a complex interplay between nutritional status and stroke outcomes (14).

The meta-analysis by Zeng et al. (15) also contributes to the discussion by assessing the impact of folic acid supplementation on homocysteine reduction and stroke risk. The analysis suggests that folic acid supplementation has a modest benefit in stroke prevention for regions without folate fortification (15). This underscores the potential influence of geographical and nutritional factors in modifying the relationship between homocysteine and stroke risk.

In conclusion, the studies presented collectively challenge the idea that elevated homocysteine levels are the sole cause of stroke. Genetic factors, nutritional interventions, and their interactions contribute significantly to stroke risk. These findings suggest a more nuanced approach to understanding stroke pathogenesis and prevention, highlighting the need for personalized interventions based on individual genetic background and nutritional status.

## 4 Preclinical studies

Investigating deficiencies in 1C in model systems has played a major role in understanding mechanisms. Impairment of 1C resulting from folate deficiency hinders the proliferation of neuroprogenitor cells in the adult hippocampus (16). When hippocampal cultures are exposed to folic acid-deficient medium, methotrexate (an inhibitor of folic acid metabolism), or homocysteine, it leads to cell death and increased susceptibility to amyloid β-peptide (Aβ)-induced cell death (17). Methyl donor deficiency causes DNA damage and uracil misincorporation, enhancing Aβ toxicity due to reduced repair of Aβ-induced oxidative DNA base modifications (17). Homocysteine triggers neuron apoptosis through a series of events, including DNA damage, PARP activation, NAD depletion, mitochondrial dysfunction, and oxidative stress (18). This suggests that homocysteine can sensitize neurons to oxidative stress and excitotoxicity, contributing to neurodegenerative disorders associated with elevated homocysteine levels. Furthermore, homocysteine activates NMDA receptors and generates free radicals, such as hydrogen peroxide, leading to cerebellar granule cell damage (19). These findings highlight the detrimental effects of impaired one-carbon metabolism and elevated homocysteine levels on neuroprogenitor cells, overall neuronal health, and the potential mechanisms involved in neurodegenerative diseases’ development.

Homocysteine promotes the proliferation of microglia and up-regulates the expression of CD11b, a marker of microglial activation (20). Brain sections from mice with hyperhomocysteinemia also show an increased number of CD11b-positive microglia. The mechanism behind this effect involves the activation of NAD(P)H oxidases, leading to the generation of reactive oxygen species (20). Homocysteine induces the phosphorylation of p47phox through the p38 MAPK pathway, up-regulating NAD(P)H oxidase activity. Inhibition of reactive oxygen species blocks microglial proliferation and activation, critical processes in the development of neurodegenerative disorders (20). These findings reveal a novel role of homocysteine in the pathogenesis of neurodegenerative diseases through its effects on microglia.

In BV-2 murine microglial cells, the combined effects of homocysteine, S-adenosylhomocysteine (SAH), and adenosine (Ado) induce significant apoptosis, as indicated by various apoptotic markers (21). This combination increases intracellular levels of reactive oxygen species (ROS) and reduces mitochondrial potential, leading to elevated caspase-9 and caspase-3 activities (21). Additionally, the combination induces intracellular hypomethylation, evidenced by decreased levels of 5-methyldeoxycytidine and altered SAM/SAH ratios (21). Pre-treatment with α-tocopherol, a potent antioxidant, reduces ROS generation and improves cell viability in the presence of SAH, homocysteine, and Ado (21). The study demonstrates that the synergistic effect of SAH, homocysteine, and Ado induces apoptosis in BV-2 cells by promoting ROS generation and intracellular hypomethylation (21).

Astrocytes exposed to high homocysteine levels undergo significant changes in their actin cytoskeleton and morphology, disrupting the glial fibrillary acidic protein (GFAP) meshwork, an essential structural component in astrocytes (22). The remodeling of the astrocyte cytoskeleton is associated with fluctuating levels of reactive oxygen species (ROS), which can be mitigated by the use of antioxidants. In contrast, neurons do not exhibit significant cytoskeletal alterations even after 24 h of homocysteine exposure (22). This study suggests that astrocytes play a protective role in response to homocysteine-induced damage by reorganizing their cytoskeleton and generating signals to ensure neuronal survival (22).

The link between elevated plasma homocysteine levels and the risk of atherothrombotic disease may be due to homocysteine acting as both a marker and a cause of oxidant stress (23). Oxidant stress is a key mediator of atherothrombotic disorders, and homocysteine could exacerbate this stress (23). Folate, a key factor in homocysteine metabolism, may play a crucial role in this process as it can act as an antioxidant, and oxidative stress, particularly due to inflammation, can deplete folate and increase homocysteine levels (23). In rabbit models, folate deficiency leads to elevated plasma homocysteine levels, and antioxidant vitamin supplementation can delay this rise. Homocysteine itself can cause cellular and macromolecular damage through oxidant mechanisms (23). In summary, homocysteine and oxidant stress may interact in a two-way street, where homocysteine can both contribute to and be a marker of oxidative injury, potentially exacerbated by folate depletion.

## 5 Discussion

Homocysteine serves as a useful marker of changes in 1C metabolism, but as the scientific evidence shows it is not the cause of disease. Over the past 90 years, research on homocysteine has revealed its relevance to diverse diseases and disorders, leading to significant advancements in diagnostic techniques and point-of-care testing (1). As technology progresses, the development of metabolomic profiles backed by large databases and artificial intelligence could allow for “point-of-care” testing, health screening, and diagnosis, with homocysteine potentially forming part of these profiles. Elevated homocysteine levels could serve as an early biomarker of disease risk, enabling early intervention and prevention strategies (1).

Precision medicine, a burgeoning discipline, aims to optimize organ function by understanding inter-individual variability in homocysteine levels due to genetically determined metabolic heterogeneity. By identifying metabolic inefficiencies, clinicians can recommend personalized diets, supplements, medical foods, or pharmaceutical interventions. Epigenetics, which is influenced by 1C metabolism, plays a crucial role in early development and lifelong health (1). Understanding the impact of homocysteine on epigenetic programming could open new avenues for “precision nutrition.”

Therapeutic supplements containing vitamin B12 and folic acid have been explored to reduce homocysteine levels, but a novel cocktail approach combining 5-methyltetrahydrofolate, methyl B12, betaine, and N-acetylcysteine (NAC) may target multiple pathways, leading to substantial homocysteine reduction and reduced associated toxicity (24). Additionally, activating the homocysteine re-methylation pathway through 5-MTHF and methyl B12 and using betaine supplementation may further improve cognitive function, especially in individuals with specific gene variants. NAC can help reduce free radical toxicities and restore intracellular GSH levels (24). Combining these components in a therapeutic cocktail could provide a potentially effective and convenient treatment, by addressing the root causes of elevated levels of homocysteine (24).

## Author contributions

SJ: Data curation, Investigation, Writing – original draft, Writing – review & editing. NJ: Conceptualization, Investigation, Supervision, Writing – original draft, Writing – review & editing.
